# Auto-phylo v2 and auto-phylo-pipeliner: building advanced, flexible, and reusable pipelines for phylogenetic inferences, estimation of variability levels and identification of positively selected amino acid sites

**DOI:** 10.1515/jib-2023-0046

**Published:** 2024-03-27

**Authors:** Hugo López-Fernández, Miguel Pinto, Cristina P. Vieira, Pedro Duque, Miguel Reboiro-Jato, Jorge Vieira

**Affiliations:** CINBIO, Department of Computer Science, ESEI—Escuela Superior de Ingeniería Informática, Universidade de Vigo, 32004 Ourense, Spain; SING Research Group, Galicia Sur Health Research Institute (IIS Galicia Sur), SERGAS-UVIGO, 36213 Vigo, Spain; 26706 Instituto de Investigação e Inovação em Saúde (I3S), Universidade do Porto, Rua Alfredo Allen, 208, 4200-135 Porto, Portugal; 26706 Faculdade de Ciências da Universidade do Porto (FCUP), Rua do Campo Alegre, s/n, 4169-007 Porto, Portugal; Instituto de Biologia Molecular e Celular (IBMC), Rua Alfredo Allen, 208, 4200-135 Porto, Portugal; School of Medicine and Biomedical Sciences (ICBAS), Porto University, Rua de Jorge Viterbo Ferreira, 228, 4050-313 Porto, Portugal

**Keywords:** Docker, pipeline maker, phylogenetics, gametophytic self-incompatibility, *Prunus S-RNase*

## Abstract

The vast amount of genome sequence data that is available, and that is predicted to drastically increase in the near future, can only be efficiently dealt with by building automated pipelines. Indeed, the Earth Biogenome Project will produce high-quality reference genome sequences for all 1.8 million named living eukaryote species, providing unprecedented insight into the evolution of genes and gene families, and thus on biological issues. Here, new modules for gene annotation, further BLAST search algorithms, further multiple sequence alignment methods, the adding of reference sequences, further tree rooting methods, the estimation of rates of synonymous and nonsynonymous substitutions, and the identification of positively selected amino acid sites, have been added to auto-phylo (version 2), a recently developed software to address biological problems using phylogenetic inferences. Additionally, we present auto-phylo-pipeliner, a graphical user interface application that further facilitates the creation and running of auto-phylo pipelines. Inferences on *S-RNase* specificity, are critical for both cross-based breeding and for the establishment of pollination requirements. Therefore, as a test case, we develop an auto-phylo pipeline to identify amino acid sites under positive selection, that are, in principle, those determining *S-RNase* specificity, starting from both non-annotated *Prunus* genomes and sequences available in public databases.

## 1 Introduction

Knowledge on the evolutionary history of a gene or gene family can highlight instances of gene duplication, providing valuable insight into the likely function of homologous/paralogous genes in different species, and adaptation at the molecular level. Hypofunctionalization (when both members of a duplicate pair experience changes in expression level such that either gene alone is not able to account for the needed gene expression, and thus both genes become essential), subfunctionalization (when each member of a gene pair loses a different but complementary function, meaning that they are now both essential genes), and neofunctionalization (when one member of the gene pair evolves a new function) are the possible fates of a gene duplicate [1]. Such knowledge is essential to understand whether the findings made for a given species can be extrapolated to other species, including humans. Inferences on the evolutionary history of a gene/gene family can also help explain morphological evolution (see for instance, [2]), and provide an explanation for why the suppression of the expression of a given gene has no apparent consequences (see for instance, [3]), among others. The genome sequence data that is available at present, already allows high quality inferences on the evolution of gene/gene families, but until 2030, the Earth Biogenome Project aims at obtaining high-quality reference genome sequences for all 1.8 million named living eukaryote species [4, 5]. Therefore, soon, life science researchers will have an opportunity to understand the evolution of any eukaryote gene with unprecedented detail. Nevertheless, there is a shortfall of bioinformaticians since the 1990 [6], which can be a major hurdle in the effective use of such a large dataset. Having easy to use bioinformatics pipelines tailored for researchers without a background in informatics is thus essential.

When performing phylogenetic analyses, it is important to show that the inferred evolutionary history does not depend on the alignment or phylogenetic method used, which usually implies the use of different software packages that may accept different input file formats. An example of such an approach is given in auto-phylo v1, where the authors describe a pipeline maker for phylogenetic studies [7]. Moreover, several filtering and checking steps are often needed to go from the download of sequence data (usually in FASTA format) to the alignment and phylogenetic inferences steps. The different software applications may require different input file formats and have different limitations, regarding, for instance, sequence header sizes, the handling of blank spaces and special characters in sequence header names, the presence of non-multiple of three sequences, or the presence of stop codons, among others. When dealing with very large datasets, the preparation of files for the different software applications can be challenging (especially for users without an informatics background), time consuming and prone to human error. In order to address these issues, software applications such as SEDA have been developed. SEDA (SEquence DAtaset builder; [8]), is an open-source, multiplatform application, with an easy-to-use graphical user interface (GUI), that can be used to perform a large number of operations on FASTA files containing DNA and protein sequences, and thus greatly simplifies this task. In order to allow the use of SEDA commands in pipelines, starting with version 1.6.0, a command line interface (CLI) is also available for SEDA. A Docker image (pegi3s/seda:1.6.0) is available for this version, at the pegi3s Bioinformatics Docker Images Project (https://pegi3s.github.io/dockerfiles/; [9]), where Docker images are available for more than 100 common bioinformatics applications and pipelines. These were instrumental to develop the auto-phylo modules [7]. Auto-phylo is distributed as a Docker image (https://pegi3s.github.io/dockerfiles/auto-phylo), and thus, in order to use it, the user only needs to install Docker. This image comes with all the advantages of the use of such containers such as being immutable and portable between computers with the same and different operating systems and versions (Linux, Windows, Mac), can be downloaded when needed and erased when no longer needed, and the installed scientific software is ready to run. When using auto-phylo, users can create automated pipelines, by simply specifying the auto-phylo modules to be used, as well as the input and output directories for each module. Therefore, even life science researchers without an informatics background can easily install and create a pipeline with auto-phylo. It should be noted that the use of Docker images, as well as applications such as auto-phylo (the pipeline and configuration files can be easily provided), can help solve the reproducibility crisis of bioinformatics analyses (see [10], for instance).

Here, we present 14 new auto-phylo modules, namely, for sequence homology search (blastn and tblastn), gene annotation (Conserved Gene Annotation, CGA), for the inclusion of reference sequences, new multiple sequence alignment modules (Mafft, Mafft_codons, Probcons, Probcons_codons, Probcons_refinement), new phylogenetic tree rooting modules (FastRoot, and Rootdigger), for the extraction of sequences associated with a given phylogenetic tree clade, for the estimation of rates of synonymous and nonsynonymous substitutions, and for the identification of positively selected amino acid sites (PSS) using either FUBAR [11], codeML [12], and omegaMap [13], thus increasing the number of operations that users can do. An application, named auto-phylo-pipeliner, with an easy-to-use GUI, is also provided, that further facilitates the creation and running of auto-phylo pipelines. As an example of the utility of the new modules, here we report the creation of three pipelines, aimed at giving insight into the evolution of the *Prunus S-RNase* gene. This example, as well as the others already presented in [7], illustrate the utility and versatility of this software application. In addition, the 10 test datasets that are provided (http://evolution6.i3s.up.pt/static/auto-phylo/v2/docs/test_data.html), further illustrate the usage of the modules.

## 2 Materials and methods

### 2.1 Auto-phylo-pipeliner development

The auto-phylo-pipeliner application here presented has been developed in Python 3.8 using Tkinter, and facilitates the preparation of the pipeline and configuration files that are needed to run the auto-phylo Docker image by providing and easy-to-use GUI. In Linux operating systems, the auto-phylo-pipeliner can be installed by invoking in the command line, the following instruction:

pip install -I -U -i https://maven.sing-group.org/repository/python-snapshots/simple auto-phylo-pipeliner

An auto-phylo-pipeliner Docker image is also given in https://hub.docker.com/r/pegi3s/auto-phylo-pipeliner.

### 2.2 Sequence data acquisition for the *Prunus S-RNase* evolution case study

In a previous work [7] we have shown how the auto-phylo Docker image can be used, to address the impact of the use of different alignment and phylogenetic inferences methods when using the same set of sequences, as well as to identify putative aldonolactone oxidoreductase sequences of bacterial origin, that may share similar biological functions with the last enzyme involved in the animal vitamin C synthesis pathway, namely, L-gulonolactone oxidase (GULO) [14]. Here, we give an additional example, on how to use auto-phylo to study the evolution of the *Prunus S-RNase* gene, using both 125 complete coding sequences (CDS) that are available at the NCBI nucleotide database (identified using tblastn (expect value of 0.05) and using as query the *Prunus avium* S12-RNase sequence (AAP92438.1); it should be noted that only sequences reporting the full CDS, and that show no ambiguous nucleotides were retained), as well as the genome sequence of 38 *Prunus* genome assemblies that are available in the NCBI Assembly GenBank database (for only four there is an annotation at the NCBI RefSeq Assembly database; those assigned with a star): *Prunus persica* (GCA_000346465.2*, GCA_024337555.1, GCA_015730445.1, GCA_018340835.1, GCA_022343065.3, GCA_020226405.1, GCA_000218175.1, GCA_000218215.1, GCA_000218195.1, GCA_019209885.1)*, Prunus dulcis* (GCA_902201215.1*, GCA_021292205.2, GCA_008632915.2), *Prunus mume* (GCA_000346735.1*, GCA_029339155.1), *Prunus salicina* (GCA_020226455.1, GCA_014863905.1, GCA_019277915.1), *Prunus mongolica* (GCA_030345395.1), *Prunus mira* (GCA_020226265.1), *Prunus fruticosa* (GCA_018703695.1), *Prunus davidiana* (GCA_020226225.1), *P. avium* (GCA_002207925.1*, GCA_013416215.1, GCA_003946875.1, GCA_012349055.1, GCA_014155035.1), *Prunus armeniaca* (GCA_903112645.1, GCA_020424065.1, GCA_020226305.1, GCA_018524995.1, GCA_903114435.1) *Prunus padus* (GCA_024362665.1), *Prunus kanzakura* (GCA_020521455.1, GCA_020521435.1), and *Prunus yedoensis* (GCA_005406145.1, GCA_002966975.2, GCA_900382725.1)).

## 3 Results

In order to understand how genes, genomes, and species evolve, phylogenetic inferences must be performed. Selecting the sequences to be included in a given study is always the first step. A gene annotation step may also be involved if the genome of at least one species to be included in the study is available but not annotated. The choice between the available multiple alignment algorithm(s) and phylogenetic inference method(s) depends on the dataset itself, since, depending on the dataset size, not all algorithms will provide results in a reasonable amount of time. Nevertheless, even when the dataset is relatively small, there is no best way of analysing the data, and the possibility that the conclusions being taken depend on the specific choices being made should always be addressed. Therefore, flexibility is a key feature when developing a pipeline with the purpose of facilitating phylogenetic analyses, and this is why auto-phylo is based on a modular system. Moreover, the estimation of variability levels and the identification of PSS can provide insight into how closely related two species/sequences are, and whether there is evidence for adaptation at the molecular level. This was the rational behind the development of the new auto-phylo modules here presented.

### 3.1 Novel auto-phylo modules

The 14 newly developed modules include gene annotation tools, further sequence homology search tools, the possibility of adding reference sequences to the dataset, further multiple sequence alignment methods, further tree rooting methods, the possibility of estimating rates of synonymous and nonsynonymous substitutions, and identify PSS (Table 1; those highlighted in bold). Sequence homology searches can now be performed using the new ‘blastn’ and ‘tblastn’ modules. The name of the query file and the BLAST expect value can be declared in the auto-phylo config file, and both modules accept as input one or multiple FASTA files, and return as output also one or multiple FASTA files containing all the sequences showing a significant hit. Moreover, MAFFT [15] (see modules ‘Mafft’ and ‘Mafft_codons’) and PROBCONS [16] (‘Probcons’, ‘Probcons_codons’ and ‘Probcons_refinement’) can now be used to align nucleotide sequences and refine alignments. Both ‘Mafft’ and ‘Probcons’ are designed to process single nucleotide FASTA files, leveraging their respective software to produce single sequence alignment FASTA files. The ‘_codons’ variants of these modules, namely ‘Mafft_codons’ and ‘Probcons_codons’, are tailored for CDS FASTA files without stop codons. These modules first translate nucleotide sequences into protein sequences using EMBOSS [17] transeq, and align the translated sequences using the respective software. Then, using TranslatorX [18], they convert these amino acid alignments back into corresponding nucleotide alignments. ‘Probcons_refinement’ was also developed to refine nucleotide sequence alignments. It accepts as input a single FASTA file containing aligned sequences and using the PROBCONS [16] program refinement option (the number of iterations to be executed is specified in the auto-phylo config file), returns a refined nucleotide sequence alignment FASTA file. The possibility to root phylogenetic trees using different methodologies is now also provided with the development of two modules, namely ‘FastRoot’ and ‘Rootdigger’, based on the software applications with the same names [19, 20]. Both modules accept Newick tree files as input and produce rooted trees in the same format as output, although ‘Rootdigger’ also needs the nucleotide sequence alignment that was used to infer the phylogenetic tree. The ‘FastRoot’ module offers three rooting methods (specified in the auto-phylo config file): Minimum Variance (MV), Midpoint Rooting (MP), and Outgroup Rooting (OG), with the latter requiring specification of an outgroup also in the config file. ‘Rootdigger’ roots the tree in either an ‘exhaustive’ or ‘early-stop’ mode, depending on the user’s preference (specified in the auto-phylo config file). A module named ‘get_phylo_taxa’ was also developed, which allows the user to extract from a Newick file all the taxa located in a given clade that is defined by two user specified sequence names in the auto-phylo config file. It requires as input the nucleotide alignment file and the tree file, which must be in the same folder, and it provides as output an unaligned FASTA file containing the corresponding nucleotide sequences. There is also the possibility to add reference nucleotide sequences (in FASTA format) to nucleotide sequence files, also in FASTA format, by using the ‘add_refs’ module. The ‘CGA’ (Conserved Genome Annotation) module belongs to the newly created Gene annotation section. This module accepts as input one or multiple nucleotide FASTA files (usually whole genomes) and implements the following steps: (1) a tblastn step to isolate the region where the gene to be annotated is located (a second tblastn can be executed using the results of the first one as database), (2) a grow sequences step to merge sequences showing a significant degree of overlap (defined by the user in the auto-phylo config file), and (3) execution of the CGA pipeline (https://hub.docker.com/r/pegi3s/cga/) in order to perform CDS annotations using as reference a single amino acid sequence provided by the user. The maximum distance between exons from the same gene, the distance around the junction point between two sequences where to look for splicing signals, the minimum size for reporting CDS and the selection model to be used must be specified in the auto-phylo config file. In order to estimate nonsynonymous (*Ka*) and synonymous (*Ks*) substitution rates by means of various models or model selection and averaging, the ‘kaks’ module was developed. This module is based on the KaKs_Calculator 2.0 [21]. It accepts as input one FASTA file containing nucleotide CDS sequences and returns a tabular formatted file containing nonsynonymous and synonymous substitution rates for each pair of sequences, as well as other additional information. Lastly, for the detection of PSS, the ‘ipssa’ module was created, that is based on the IPSSA software application [22]. This module is used to automatically identify PSS and check if there is evidence for recombination in the sequence data. It accepts as input one CDS FASTA file, and returns a tabular formatted file containing the results of all selected PSS methods. In the auto-phylo config file users can specify the number of sequences to be analysed, the alignment method to be used (ClustalW [23] and MUSCLE [24]), and the algorithms of choice to infer PSS, namely FUBAR [11], codeML [12], or omegaMap [13], as well as the parameters required by these methods.

**Table 1:** Auto-phylo modules according to general purpose. Modules highlighted in bold are new in auto-phylo version 2 (for details see the auto-phylo manual at http://evolution6.i3s.up.pt/static/auto-phylo/v2/docs/overview.html).

Purpose	Modules
Blast	**blastn**; **tblastn**; tblastx
FASTA processing	add_taxonomy; check_contamination; disambiguate; CGF_and_CGA_CDS_processing; merge; prefix; prefix_out; remove_stops
Sequence alignment (produces a *.nuc_alignment)	Clustal_Omega; Clustal_Omega_codons; **Mafft**; **Mafft_codons**; **Probcons**; **Probcons_codons**; **Probcons_refinement**; T-coffee; T-coffee_codons;
Tree building (produces a *.nwk)	**add_refs**; **FastRoot**; Fasttree; **get_phylo_taxa**; me_tree; ml_tree; mp_tree; MrBayes; nj_tree; **Rootdigger**; tree_collapse; upgma_tree
Model checking	JModel_test
Gene annotation	**CGA**
Divergence	**kaks**
Positive selection	**ipssa**
Special	split

In total there are now 37 modules in auto-phylo v2 that can be selected according to the user needs (Table 1; grouped by their general purpose; for further details on each module see the auto-phylo manual at http://evolution6.i3s.up.pt/static/auto-phylo/v2/docs/overview.html). As the previously developed modules, the new ones are based on the pegi3s Bioinformatics Docker Images Project [9] images, as well as on the CLI recently made available for the SEDA v1.6.0 [8] software. In order to avoid out of memory errors when processing a large number of files, it is advisable, if possible, to use the split option. This special command takes as input a single argument, namely, the number of groups to consider, and is invoked after a regular command [7]. For instance, the instruction: “tblastx my_data_dir out_dir split 25” will split the files that are in the “my_data_dir” directory into 25 equal size subfolders. The data on each subfolder will be processed independently, but the output of all independent analyses will still be saved in the same “out_dir” directory.

### 3.2 Auto-phylo-pipeliner

**Figure 1:**
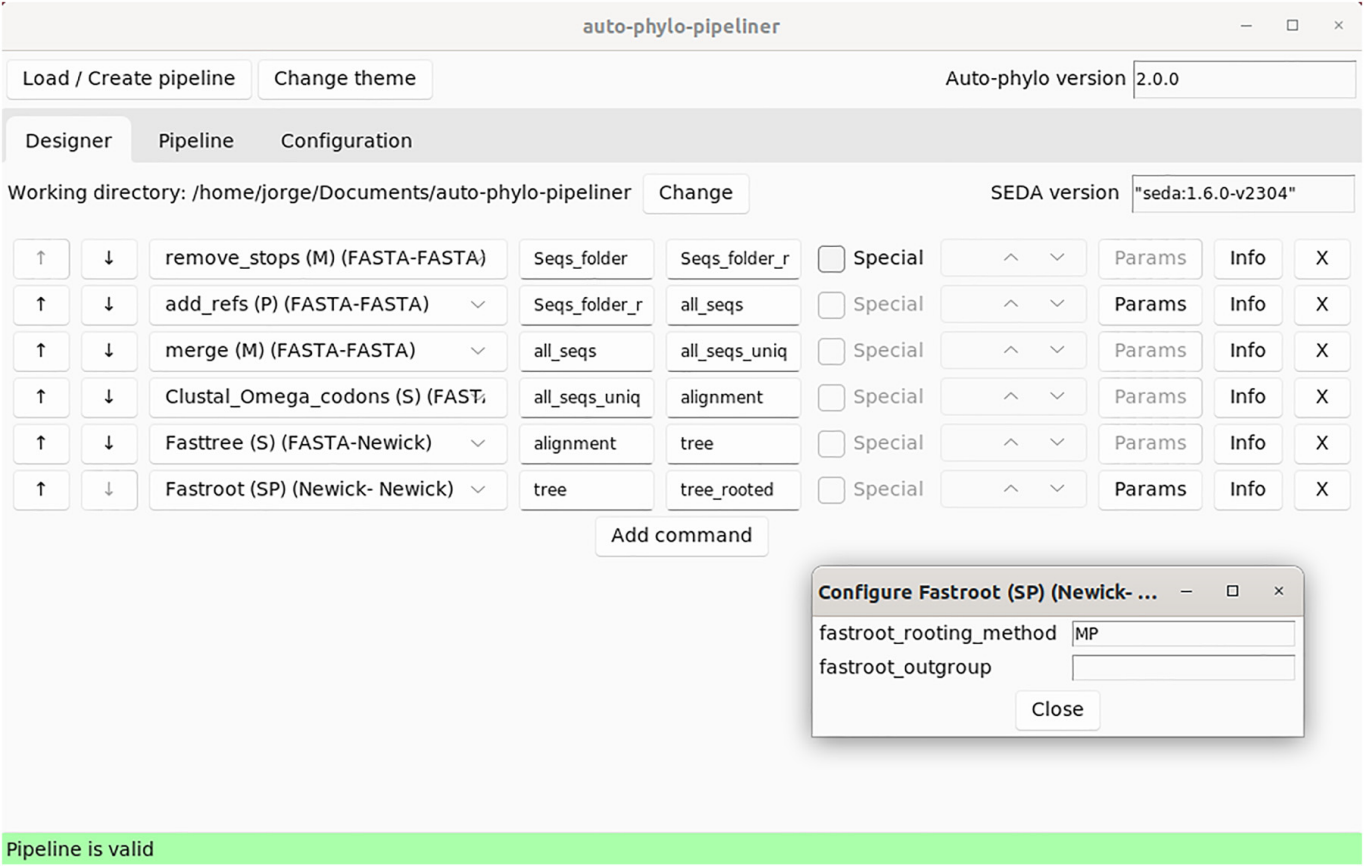
Interface of the auto-phylo-pipeliner software application loaded with the pipeline 2 configuration used to study the evolution of the *Prunus S-RNase.*

Although users can still use the auto-phylo Docker image to build pipelines, in order to avoid errors while preparing the pipeline and configuration files, users are advised to use the auto-phylo-pipeliner application here presented, that offers a graphical interface for the preparation of pipeline and configuration files (Figure 1).

When using the auto-phylo-pipeliner application, the first thing to do is to select the working directory using the “Load/Create pipeline” button. If a file named “config” and another one named “pipeline” are found in the working directory, the user may choose to load them. Changes can be made to the two files in the “Designer” tab, but in order to be written to the “config” and “pipeline” files, they must be validated by looking at the “Pipeline” and “Configuration” tabs. If no such files are present, they are created containing the minimum required information. Then, the user should select (from left to right) the desired auto-phylo module, the input directory, the output directory, whether to use or not the special “Split” function (this option is disabled if the module is not compatible with this option), and specify the needed parameters (this option is disabled if there is no need to specify any parameters). The “Info” button redirects the user to the section of the auto-phylo manual where the module is described. At the left, the arrows allow the reordering of the actions to be performed, while the cross at the right allows the deletion of a given action. For each module, the values within the first set of brackets mean that the module accepts a single (S), or multiple (M) files. P means that in order to use the module some parameters must be specified. The values within the second set of brackets refer to the type of file that is accepted and the one that is produced. It should be noted that it is possible to declare branched pipelines, where the input directory of the next operation is not the same as the output directory of the previous operation. When the pipeline design is finished, the user can run the auto-phylo Docker image using the specified “config” and “pipeline” files by invoking the run.sh (that is generated automatically by auto-phylo-pipeliner) command on the command line.

As stated in [7], intermediate files, which are produced during the processing of the data, may contain information that is potentially relevant. The file named “blocks_and_commands” indicates where such intermediary files have been saved.

The development of new modules for auto-phylo often require only basic knowledge on how to build a Docker image for the required software application and basic knowledge in Bash, as explained in [7], and in the auto-phylo manual (http://evolution6.i3s.up.pt/static/auto-phylo/docs/script.html). The custom modules can be imported into the pegi3s auto-phylo Docker image by invoking the “run -it” Docker option, and once inside the Docker image, copying the custom module to the same folder where all other modules are, do the needed changes in the file named “main”, and then using the Docker “save” command, to save the new state of the Docker image. Alternatively, by using “git clone https://github.com/pegi3s/auto-phylo” the user can clone the project, change the Dockerfile to include one more “COPY” instruction followed by the custom module name, do the needed changes in the file named “main”, and build an image using the Docker “build” option.

### 3.3 The study case: *Prunus S-RNase* evolution

Gametophytic self-incompatibility (GSI), is a common pre-zygotic self-incompatibility genetic system in eudicots, that allows the recognition and rejection of self-related pollen by the pistil, thus avoiding fertilization of plants with the same *S*-haplotype [25]. In *Prunus* (Rosaceae), GSI specificity is determined by two linked genes, one that encodes the *S*-pistil gene, a protein with ribonuclease activity, called *S-RNase*, that is expressed in the pistil, and the other that encodes for an *S*-haplotype-specific F-box protein (*SFB*s) that is specifically expressed in pollen (see [26] for a review). So far, this is the only genus with a self-recognition mechanism, where the two *S*-pollen genes show patterns of co-evolution [27–30]. In order to show the utility of auto-phylo and auto-phylo-pipeliner, we have used these tools to address the evolution of the *Prunus S-RNase* gene.

**Figure 2:**
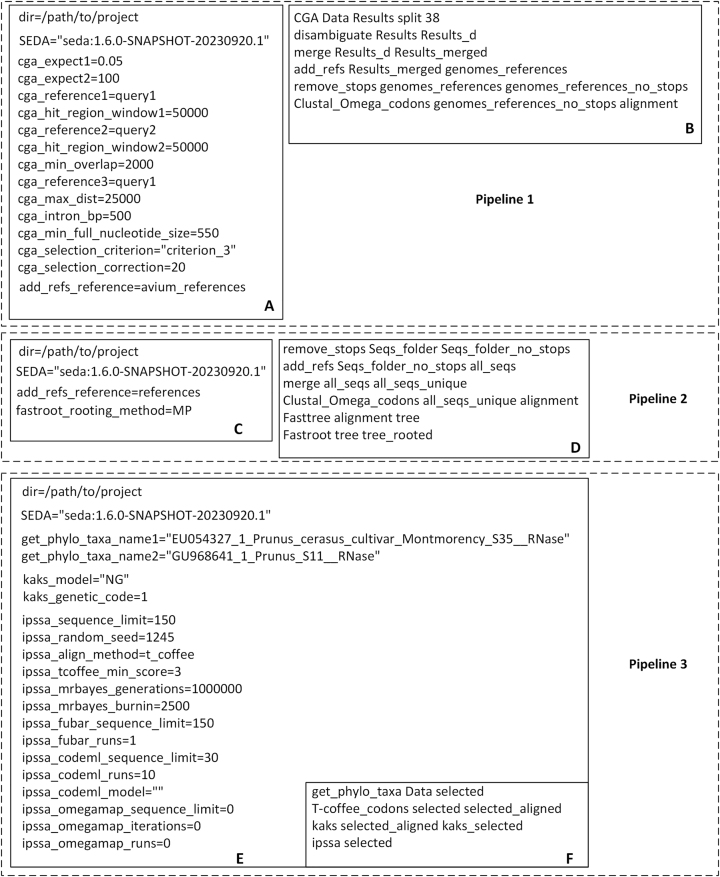
Settings of the three auto-phylo pipelines used to address the *Prunus S-RNase* evolution. In the first pipeline (A and B, config and pipeline files, respectively), the auto-phylo Conserved Gene Annotation (CGA) module is used to annotate putative complete *S-RNase* CDSs using a reference protein sequence and non-annotated genomes, and to produce a nucleotide sequence alignment. In the second pipeline (C and D, config and pipeline files, respectively) a phylogeny is produced to establish whether all *S-RNase* CDS sequences identified in pipeline 1, are indeed *S-RNase* sequences. In the third pipeline (Figure 2E and F for “config” and “pipeline files”, respectively) pairwise *Ks* (the rate of synonymous substitutions per synonymous site) and *Ka* (the rate of non-synonymous substitutions per non-synonymous site) values are estimated, and amino acid sites under positive selection are identified.

As a first step, we have collected 125 full CDS *S-RNase* sequences from the NCBI nucleotide database. Nevertheless, there are also 38 *Prunus* genomes, of which only four are annotated, and thus, the analysis of the 38 *Prunus* genomes could reveal novel *S-RNase* CDS sequences. Therefore, the main goal of the first pipeline (Figure 2A and B for “config” and “pipeline” files) is to perform an annotation of the 38 *Prunus* genomes, using the auto-phylo Conserved Gene Annotation (CGA) module, and one *Prunus* S-RNase protein sequence as reference (AAP92438.1 from *P. avium*). The pipeline implements all needed operations to annotate *S-RNase*-like sequences (Figure 2B), including a first step to isolate the regions of interest using protein to translated nucleotide homology (those where a significant tblastn hit is obtained, in our case, with a maximum size of about 100 Kb), a further selection of the regions of interest, using just the protein region that corresponds to the small and less conserved, first encoded *S-RNase* exon (MGMLKSSLAFLVLAFAFFLCFIMSAGD), followed by the annotation of those regions by CGA, using the initial tblastn protein query as reference. A disambiguation step is then performed to ensure that all annotated CDS sequences have a different name in order to comply with the standard rules for FASTA files. Then a merging step (that includes the removal of identical sequences) is performed followed by a step where reference sequences (a subset of 12 *P. avium* sequences present in the 125 complete CDS *S-RNase* sequences file), are added to the resulting file. Then, stop codons are removed, CDS sequences are translated, and aligned using Clustal Omega, and the resulting alignment used as a guide to obtain the corresponding nucleotide alignment (Clustal_Omega_codons module). It should be noted that CGA is a greedy algorithm, and thus, in order to avoid out of memory issues, we have used the split command in order to make sure that only one genome is being processed at any time.

For only 11 out of the 38 *Prunus* genomes, it was possible to get at least one CDS annotation for an S-RNase-like CDS sequence (Supplementary Figure 1). Nevertheless, only one protein reference sequence was used, as well as a single set of parameters. Therefore, in order to be sure that no *S-RNase* sequences have been missed, this pipeline should be re-run with more reference sequences and different parameter sets. Moreover, the use of different CGA criterions (here we have used criterion 3) may result in different annotations. These issues should be taken into account if the purpose of the work is to get the best possible annotation of *S-RNase* sequences in every genome. In this work, the main objective was to quickly determine whether *Prunus* genomes encode or not novel *S-RNases*, which only required a first attempt to annotate the *S-RNase* gene. A better annotation could be obtained later on if the *S-RNase* alleles are indeed novel. It should be noted that there is already evidence that a better annotation could have been obtained. For instance, the inspection of the alignment generated at the end of this first pipeline already reveals that one of the annotated sequences is missing the first *S-RNase* exon (the one annotated on scaffold CM035387.1; Supplementary Figure 1), two others include in-frame intron sequence (the ones annotated on scaffold LR694010.1; Supplementary Figure 1), and in a third case the second intron is 24 bp longer than it should be (the one annotated on scaffold LR694011.1; Supplementary Figure 1). Because of its impact on the remaining analyses, the former sequence was removed, and the ones that include in-frame intron sequence were corrected by hand.

The aim of the second pipeline (Figure 2C and D for “config” and “pipeline” files, respectively) is to establish whether all *S-RNase* CDS sequences being considered (those coming from the genomes, as well as those collected from the NCBI nucleotide database) are indeed *S-RNase* sequences. This is relevant, since in *Prunus*, there are other genes that share 20–30 % sequence identity with the *S-RNase,* such as the *PA1* gene ([31]; Supplementary Figure 2). Therefore, the second pipeline implements all needed steps to remove stop codons from the sequences collected from NCBI nucleotide database, adds to the resulting file the trusted CDS coming from the *Prunus* genome annotations, aligns the translation of the CDS sequences using Clustal Omega [32], and uses the resulting alignment to obtain the corresponding nucleotide alignment (“Clustal_Omega_codons” module), infers a maximum likelihood tree using Fasttree [33], and roots (using middle point rooting) the resulting tree using Fastroot [19] (Figure 2D). This analysis reveals that two of the sequences coming from the genomes were not *S-RNases* (Supplementary Figure 2). This evidence, together with the observation that the *S-RNase* CDS sequences that could be annotated are spread all over the phylogeny (Supplementary Figure 2), suggest that the ability to annotate only 11 out of the 38 *Prunus* genomes is likely more related to the coverage of the genomes, rather than limitations of the CGA algorithm, although in some cases the latter could be true. Knowledge on the genetic basis of specificity determination is needed for a better management of fruit production and germplasm selection, since in cultivated self-incompatible species, information on sexual compatibility determines fruit set. Cultivars carrying different *S*-alleles (belonging to different cross-incompatibility groups) must be inter-planted in orchards. Thus, this information is needed for orchard planning and appropriate crosses in breeding programs [34, 35]. In the last two decades, the cloning and characterization of the *Prunus S*-locus genes allowed the design of degenerate primers for conserved exonic regions within the *S-RNase* and *SFB* genes. Fast and accurate PCR methods for *S*-allele typing based on the polymorphism length of amplified fragments have been widely used [36–42]. Nevertheless, in this methodology, previous knowledge on the *S*-allele sequences is required. With the lowering of genome sequencing costs, it is, in principle, now feasible to use genome sequence data for *S-RNase* identification, in which case workflows similar to the pipelines 1 and 2 here presented could be useful. Furthermore, predicting *S-RNase* specificities from sequencing data, using information on the amino acids under positive selection, can be useful, since closely related *S*-RNases could represent different specificities, and the crosses that need to be performed to establish whether they are indeed different specificities, are in many cases not possible. Thus, the third pipeline (Figure 2E and F for config and pipeline files, respectively) implements all needed operations to calculate pairwise *Ks* (the rate of synonymous substitutions per synonymous site) and *Ka* (the rate of non-synonymous substitutions per non-synonymous site) values, and identify amino acid sites as being under positive selection using FUBAR [11] and codeML [12], as implemented in IPSSA [22]. Therefore, the pipeline starts by getting all sequences that in the tree obtained in the previous pipeline are in between the two most external sequences of interest. The auto-phylo “ipssa” module is used to process the resulting file, and provide a list of PSS, using two methods, namely FUBAR and codeML (models 1, 2, 7 and 8). This pipeline also performs, the translation of the selected sequences from the tree and aligns them using the T-coffee [43] algorithm. The resulting alignment is used as a guide to obtain the corresponding nucleotide alignment (“T-coffee_codons”). The latter is used to calculate *Ka* and *Ks* values using the Nei-Gojobori method (Figure 2F).

**Figure 3:**
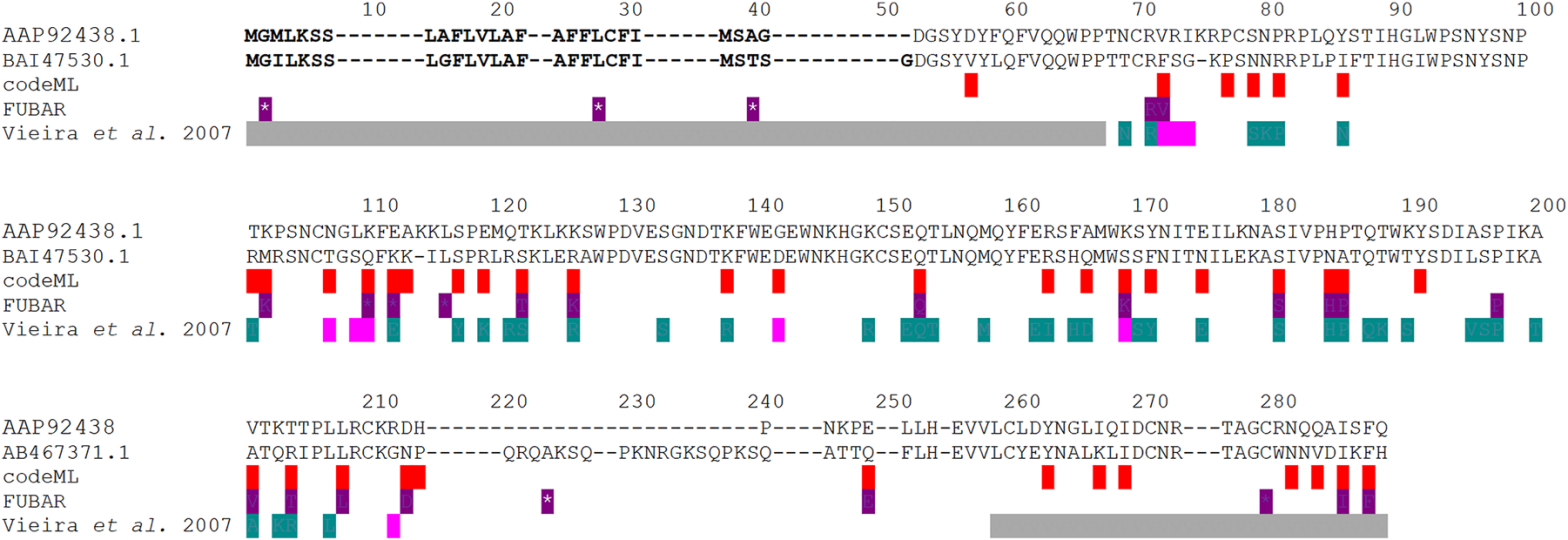
Summary of the identification of positively selected amino acid sites (PSS. For FUBAR, 125 complete CDS sequences were used, while for codeML, 10 replicas of 30 randomly extracted CDS sequences were used). The results are shown using sequences AAP92438.1 and BAI47530.1 as protein references relative to the translated CDS sequences results. PSS highlighted in red have been identified using codeML and those highlighted in purple have been identified by FUBAR (those with a star indicate positions that in the sequence alignment show alignment gaps with a frequency higher than 14 %). For comparative purposes the PSS identified in [29] are also shown (in pink the positions with confidence values above 95 % and in green the amino acid positions with confidence values between 50 and 95 %). The region highlighted in bold is the peptide signal. Gray regions are those not analysed in [29].

There are 18 PSS in common when using FUBAR and codeML (Figure 3). Of these, 11 had been already identified in [29], when using a set of 88 partial CDS sequences. There are also 23 PSS that are only identified by codeML. These are not necessarily false positives, since 11 had been already identified [29]. Finally, there are eight PSS that are only identified by FUBAR. Three of these are located in the predicted peptide signal region (inferred using SignalP – 5.0; https://services.healthtech.dtu.dk/services/SignalP-5.0/) that does not have a role in specificity determination, and thus are likely false positives. This number is not far away from the prediction of two false positive PSS made by FUBAR. Of the remaining five PSS, two were already identified [29]. The percentage of PSS that have been previously identified is not significantly different among the different categories (*P* > 0.05 for every pairwise comparison). It should be noted that the full CDS sequences here used only represent 21.6 % of the specificities used in [29]. Even when assuming that sequences with up to three amino acid differences code for the same specificity, only 47.7 % of the specificities used in [29] are here represented. This may help explain why not all PSS identified in [29] are here identified as well.

**Figure 4:**
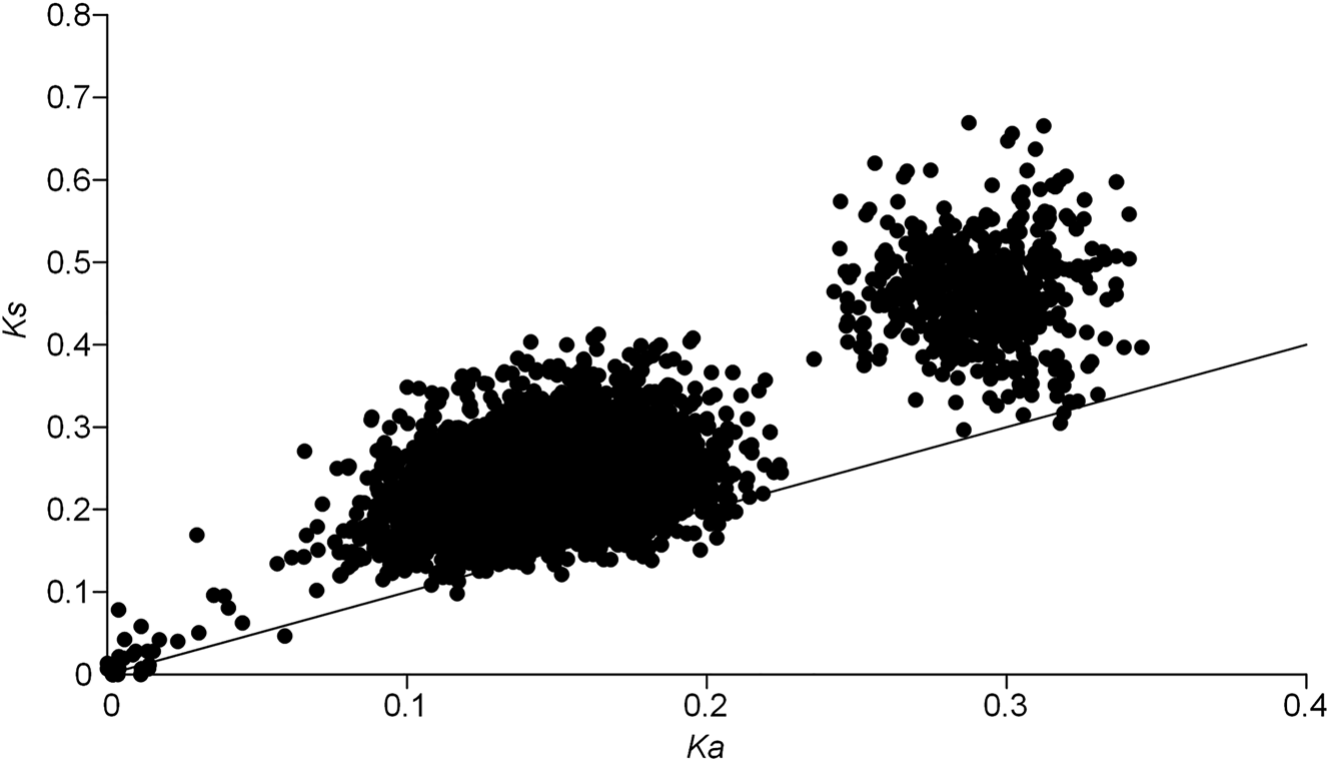
Plot of the rate of synonymous substitutions per synonymous sites (*Ks*) and the rate of non-synonymous substitutions per non-synonymous sites (*Ka*). Below the line are *Ka*/*Ks* > 1, an indication of positive selection.

The results of the *Ka* and *Ks* pairwise calculations are graphically illustrated in Figure 4. There are three clear clusters, namely, one comprising very similar alleles (with a *Ks* value below 0.1), another one involving the comparison of divergent alleles (with a *Ks* value in between 0.1 and 0.35), and a third one that includes comparisons of very divergent alleles (with *Ks* values above 0.35). The frequency of comparisons for which a *Ka*/*Ks* value above one is obtained (a classical sign of positive selection) is very different in the three groups, namely 23.1 % (6 out of 26), 2.2 % (86 out of 3840), and 0 % (0 out of 692). The differences are highly significant for every one of the possible comparisons involving two out of the three groups (*P* < 0.00005 in every case). This is the opposite of what should be expected, given that, for comparisons involving very divergent alleles, the *Ks* value might be underestimated due to the occurrence of multiple substitutions at the same site, which could lead to an inflated estimate of the *Ka*/*Ks* value. Therefore, even very similar sequences might represent different specificities, that did not yet age enough (accumulated enough neutral synonymous mutations) to produce a *Ka*/*Ks* value below one.

In conclusion, the use of three easy-to-build (especially when using the auto-phylo-pipeliner GUI here presented) auto-phylo pipelines, that can be re-run with the same (for repeatability purposes) or new data (for comparative purposes), allowed the retrieval of novel full length *S-RNase* CDS sequences from non-annotated *Prunus* genomes, the identification of novel PSS (as well as the confirmation of a fraction of those already reported), and gave insight into the first steps of *S-RNase* specificity evolution. Once a pipeline is defined, in principle, it will run without problems with the same type of data, especially if it comes from the same database, meaning that the auto-phylo pipelines here developed can be used to analyse similar problems in species other than *Prunus*. Although the available auto-phylo modules already allow a great degree of flexibility when building a pipeline, new modules keep being added to auto-phylo, thus further increasing the auto-phylo usefulness.

## 4 Conclusions

The new auto-phylo v2 provides 14 new modules for different purposes, thus enhancing the user’s capability of building advanced, flexible, and reusable custom pipelines for phylogenetic analyses, estimation of variability levels, and identification of PSS. One module for gene annotation is now also provided giving the user the possibility to use non-annotated genomes in their inferences. Distributed as a Docker image, the auto-phylo-pipeliner software further facilitates the creation and execution of complex automated pipelines, streamlining high-quality phylogenetic analyses. Furthermore, if the configuration, pipeline files, and data to be used are made accessible, the analyses can be effortlessly replicated. Beyond the two biological examples provided in [7], three additional use-cases for auto-phylo are presented. These illustrate the ease with which the tool can offer insights into biological challenges addressable through sequence data, even when handling large datasets or when performing complex analyses.

## Supplementary Material

Supplementary Material Details
