# Supplementary material for: Auto-phylo v2 and auto-phylo-pipeliner: building advanced, flexible, and reusable pipelines for phylogenetic inferences, estimation of variability levels and identification of positively selected amino acid sites
Source: J Integr Bioinform. 2024 Mar 27;21(2):20230046. doi: 10.1515/jib-2023-0046 (PMC11378518; doi:10.1515/jib-2023-0046)
Supplement: Supplementary file 1 — Supplementary Material Details [file j_jib-2023-0046_suppl_001.docx]

Supplementary Figure 1. Nucleotide alignment obtained using pipeline 2 ([Figure 2C and D](#fig2)) and the *Prunus S-RNases* obtained using pipeline 1 ([Figure 2A and B](#fig2)) starting from non-annotated *Prunus* genomes, as well as the 12 retrieved *P. avium S-RNases*. In bold are those sequences that include an in-frame intron sequence, underlined is the annotated sequence that misses the first *S-RNase* exon, in italics the sequence that has a longer intron than expected, and with stars the sequences that are very divergent and that, by performing phylogenetic analyses, are non-*S-RNases*. In bold and underlined are the putative splicing sites used to remove the in-frame intron sequences.

Supplementary Figure 2. Phylogenetic tree of the sequences obtained in pipeline 1 (marked in blue), after manual correction of the in-frame intron regions (as shown in [Supplementary Figure 1](#figS1)), the 125 *Prunus S-RNases* complete CDS sequences from GenBank, *P. avium PA1*, and *Malus* and *Pyrus S-RNases*. The tree was mid-point rooted.
